# Supplementary material for: Efficacy and Safety of Digital Single-Operator Cholangioscopy in the Diagnosis of Indeterminate Biliary Strictures by Targeted Biopsies: A Systematic Review and Meta-Analysis
Source: Diagnostics (Basel). 2020 Sep 2;10(9):666. doi: 10.3390/diagnostics10090666 (PMC7555631; doi:10.3390/diagnostics10090666)
Supplement: Supplementary file 1 [file diagnostics-10-00666-s001.pdf]

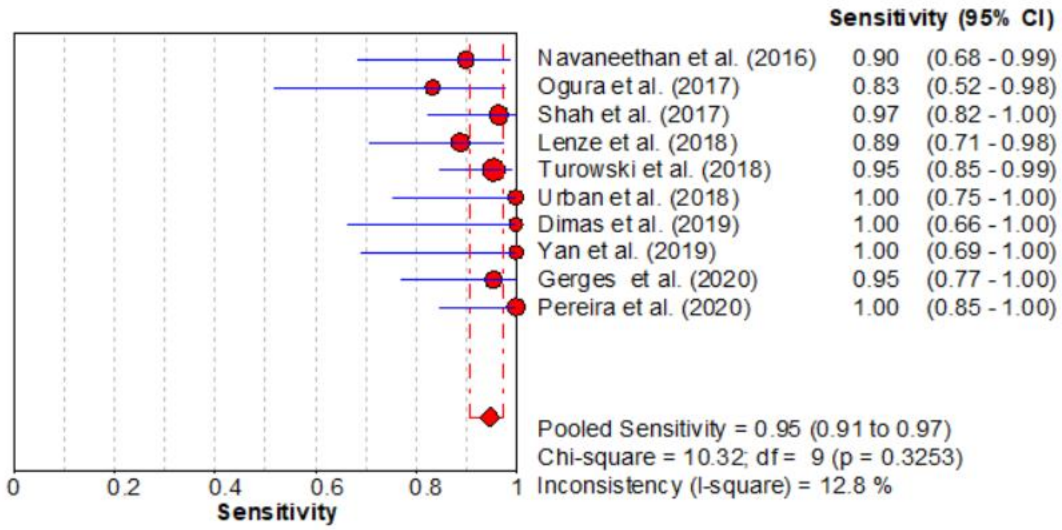

(**a**)

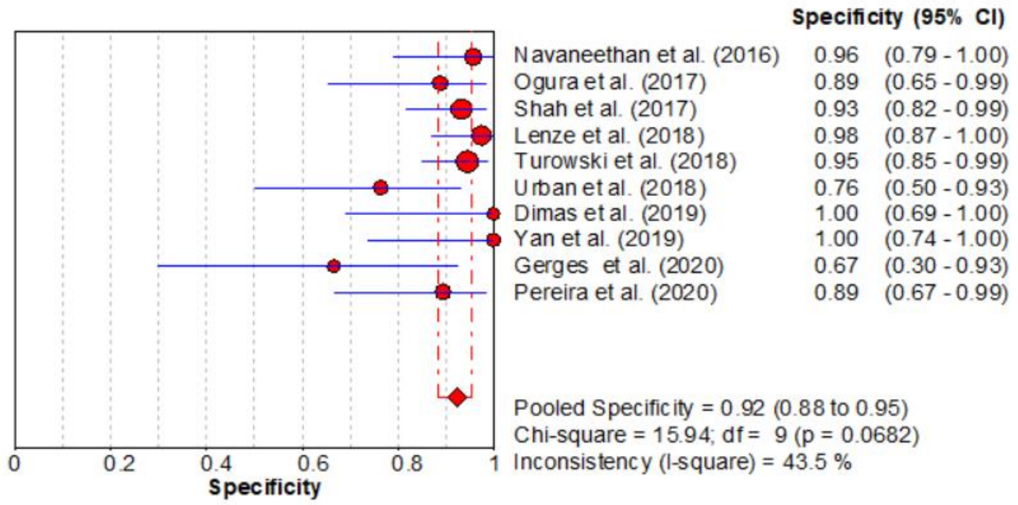

(**b**)

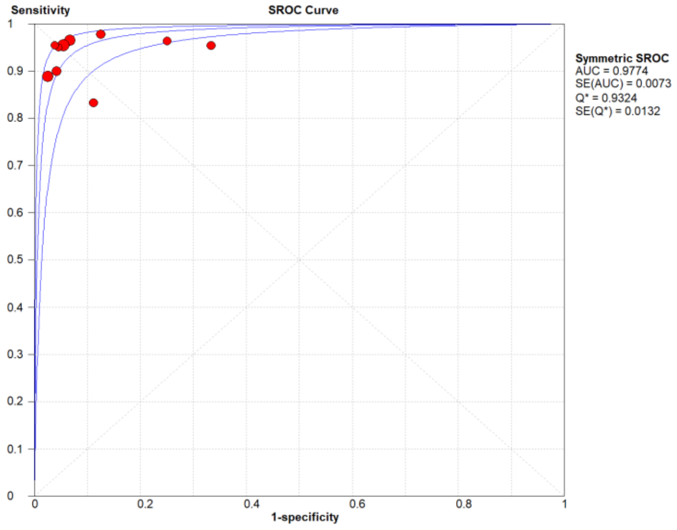

(**c**)

Figure S1. The forest plot of studies that reported the diagnostic yield of DSOC by VI. (**a**) The pooled

sensitivity for IBDS was 0.95 (95% CI: 0.91–0.97); (**b**) The pooled specificity for IBDS was 0.92 (95% CI: 0.88–0.95); (**c**) The summary receiver's operative characteristics (SROC), with an area under the curve (AUC) of 0.9774.
